# Human cytomegalovirus: taking the strain

**DOI:** 10.1007/s00430-015-0411-4

**Published:** 2015-04-17

**Authors:** Gavin W. G. Wilkinson, Andrew J. Davison, Peter Tomasec, Ceri A. Fielding, Rebecca Aicheler, Isa Murrell, Sepher Seirafian, Edward C. Y. Wang, Michael Weekes, Paul J. Lehner, Gavin S. Wilkie, Richard J. Stanton

**Affiliations:** Department of Medical Microbiology, Henry Wellcome Building, Cardiff University School of Medicine, Heath Park, Cardiff, CF14 4XN UK; Cambridge Institute for Medical Research (CIMR), Wellcome Trust/MRC Building, Addenbrooke’s Hospital, Hills Road, Cambridge, CB2 0XY UK; MRC – University of Glasgow Centre for Virus Research, Sir Michael Stoker Building, 464 Bearsden Road, Glasgow, G61 1QH UK

**Keywords:** Cytomegalovirus, Natural killer cells, Genomics, Proteomics

## Abstract

In celebrating the 60th anniversary of the first isolation of human cytomegalovirus (HCMV), we reflect on the merits and limitations of the viral strains currently being used to develop urgently needed treatments. HCMV research has been dependent for decades on the high-passage strains AD169 and Towne, heavily exploiting their capacity to replicate efficiently in fibroblasts. However, the genetic integrity of these strains is so severely compromised that great caution needs to be exercised when considering their past and future use. It is now evident that wild-type HCMV strains are not readily propagated in vitro. HCMV mutants are rapidly selected during isolation in fibroblasts, reproducibly affecting gene RL13, the UL128 locus (which includes genes UL128, UL130 and UL131A) and often the U_L_/*b*′ region. As a result, the virus becomes less cell associated, altered in tropism and less pathogenic. This problem is not restricted to high-passage strains, as even low-passage strains can harbour biologically significant mutations. Cloning and manipulation of the HCMV genome as a bacterial artificial chromosome (BAC) offers a means of working with stable, genetically defined strains. To this end, the low-passage strain Merlin genome was cloned as a BAC and sequentially repaired to match the viral sequence in the original clinical sample from which Merlin was derived. Restoration of UL128L to wild type was detrimental to growth in fibroblasts, whereas restoration of RL13 impaired growth in all cell types tested. Stable propagation of phenotypically wild-type virus could be achieved only by placing both regions under conditional expression. In addition to the development of these tools, the Merlin transcriptome and proteome have been characterized in unparalleled detail. Although Merlin may be representative of the clinical agent, high-throughput whole-genome deep sequencing studies have highlighted the remarkable high level of interstrain variation present in circulating virus. There is a need to develop systems capable of addressing the significance of this diversity, free from the confounding effects of genetic changes associated with in vitro adaptation. The generation of a set of BAC clones, each containing the genome of a different HCMV strain repaired to match the sequence in the clinical sample, would provide a pathway to address the biological and clinical effects of natural variation in wild-type HCMV.

## Introduction

Margaret Smith originally described the propagation in vitro of viruses from two neonates who succumbed to cytomegalic inclusion disease (CID) in 1954, although publication of this work was delayed because of unwarranted concerns about potential contamination with murine cytomegalovirus (MCMV) [1, 2]. Thomas Weller isolated the Davis strain fortuitously when culturing a liver biopsy with embryonic muscle cells from a patient with suspected toxoplasmosis, before growing strains Esp and Kerr from patients with CID [3]. The agent, which was eventually named human cytomegalovirus (HCMV), was thus already connected with CID. Wallace Rowe also independently isolated HCMV while propagating adenoid tissue in vitro, with three cultures undergoing spontaneous degeneration due to an infection that exhibited characteristic intranuclear inclusion bodies. This virus, designated Ad. 169, grew though cultures of adenoid tissue taken from a 7-year-old girl. Rowe provided serological evidence that strains Ad. 169, Smith and Davis were closely related and showed that seroprevalence to HCMV increased gradually with age to >80 % of the population [4]. Thus, these pioneering studies not only established the tools necessary to study this pathogen, but also demonstrated that HCMV was widespread in the community and associated with CID.

Rowe’s prototype virus, subsequently referred to as strain AD169, was taken up by laboratories worldwide and became a workhorse of HCMV research. Twenty-six years later, sub-genomic DNA clones encompassing the complete AD169 genome were utilized to generate restriction endonuclease cleavage maps, and these in turn powered pioneering investigations into HCMV transcriptional regulation, gene expression and sequence analysis [5–9]. Progress in molecular virology was spurred on by competition between groups using strains AD169 and Towne; Towne was initially developed as an attenuated vaccine by passaging 125 times in vitro [10].

HCMV research was transformed by access to the complete sequence of strain AD169, which at the time was the longest contiguous segment of sequenced DNA [11]. Comparative analyses showed that HCMV contains a subset of core genes that have homologues in all herpesviruses and that a large proportion of the viral genome was derived from extensive duplication of homologous gene families that are generally restricted to human and simian CMVs. Only 26 % of HCMV canonical genes (45/171) are essential for viral replication in vitro [12, 13]. We are particularly interested in the contribution made by the other 74 % in promoting virulence in vivo.

## Genetic changes to laboratory strains

**Fig. 1:**
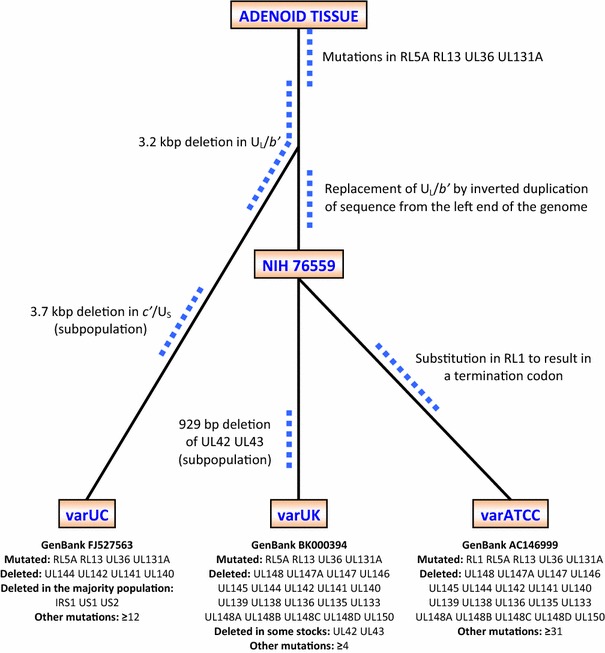
Evolution of genetic changes that have accumulated in the most commonly used variants of strain AD169 (varUK and varATCC) and a variant obtained from the University of Chicago (varUC) that retains a part of the U_L_/*b*′ region. Adapted from figure originally published in the *J. Gen. Virol.* [15], reusing the author’s own content

Characterization of the HCMV genome brought much greater resolution to studies of gene usage and function. However, a key study revealed that AD169 and Towne had each suffered substantial deletions (15 and 13 kb, respectively) of a sequence at the right end of the long unique region (U_L_) that is designated U_L_/*b*′, combined with a compensating expansion of the long terminal repeat, TR_L_/IR_L_ [14]. The genetic integrity of AD169 was subsequently subjected to a systemic evaluation that compared three variants: one sequenced in Cambridge (varUK; sourced from St George’s Hospital, London), a second distributed by the American Type Culture Collection (varATCC) and a third obtained from the University of Chicago (varUC) that has a less extensive deletion of the U_L_/*b*′ region than the other two variants (Fig. 1). The analysis clearly revealed that issues with all three variants extend beyond the loss of all or part of the U_L_/*b*′ region, as numerous genetic changes had accumulated during extensive passage of this strain since its initial isolation. Moreover, the variants had clearly diverged during their passage in various laboratories (Fig. 1). A comparable situation also exists with Towne [15].

Our misgivings concerning the validity of using AD169 and Towne as model strains to investigate HCMV pathogenesis reached a tipping point with the publication of a study on the UL36 gene. In a German variant of AD169 (varDE), UL36 was shown to be an efficient inhibitor of caspase 8, yet a single amino acid substitution (C131A) in varATCC and varUK ablates this function [16]. Thus, a point mutation had been selected in vitro that completely abrogates a viral function that is counter to a key immune defence (apoptosis), but the loss of gene function was not obvious from the UL36 sequence. The clear concern was that any HCMV gene in any cultured viral stock could be mutated, and this fact could go unrecognized.

## Rapid selection of mutations in low-passage strains

A prospective study undertaken by Dargan and co-workers revealed that clinical viruses change in a reproducible manner when cultured in vitro. Fibroblasts, epithelial and endothelial cells were infected in parallel with three low-passage HCMV strains (passage 4–5), and the infected cell cultures were then passaged weekly 50–63 times, before sequencing the complete genome of each passaged strain and comparing it with the original clinical sample at selected loci [17]. Changes were observed in all viruses. Mutations were selected first in gene RL13 (passage 8–16), then in either gene UL128, UL130 or UL131A (the UL128 locus, UL128L) (passage 15–20), and, in some cases, eventually in U_L_/*b*′, focusing on the gene UL140–UL145 region (passage 32–63) [17]. Sporadic mutations also occurred in other regions. Although HCMV mutants were selected in all cell lines tested, defects in UL128L were specifically associated with fibroblast culture, a phenomenon noted previously in other passaged strains [18, 19]. Nevertheless, the overall picture was clear. All HCMV isolates cultured from clinical samples were “genetically unstable in all cell types tested”. The outgrowth of mutants appears to be inevitable and rapid [17].

## Requirement to define wild-type HCMV gene usage

**Fig. 2:**
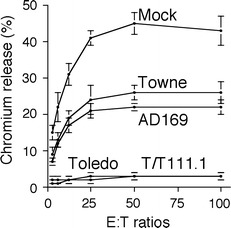
Low-passage strain Toledo provides more effective protection against NK cells than either of the laboratory strains AD169 or Towne. An NK cytolysis assay performed in which an NK cell line (NKL) was incubated with human foetal foreskin fibroblasts infected for 72 h with the HCMV strain indicated. HCMV T/T11 1.1 is a version of Towne into which the U_L_/*b*′ region from Toledo has been inserted. The proportion of target cells lysed by the NK cell line was measured by the release of radioactive chromium (^51^Cr). Adapted from a figure originally published in *Nature Immunology* [27], reusing the author’s own content

Cytotoxic T cells recognize viral peptides presented on the cell surface by endogenous MHC-I molecules. The proteins encoded by HCMV genes US2, US3, US6 and US11 act in concert to prevent newly synthesized MHC-I reaching the cell surface, thereby protecting virus-infected cells from cytotoxic T cells [20]. However, endogenous MHC-I molecules also serve as the chief ligands for NK cell inhibitory receptors. Downregulation of MHC-I from the surface thus renders HCMV-infected cells more vulnerable to NK cell attack [21]. NK cells play a critical role in controlling herpesvirus infections, and individuals with defects in their NK cell response are particularly vulnerable to HCMV disease [22]. NK cells constitute a heterogenous population that differentially express a diverse range of activating and inhibitory receptors and are capable of detecting and killing virus-infected targets [23]. Even though MHC-I is efficiently downregulated by HCMV, cells infected with a low-passage strain (e.g. Toledo) exhibit remarkable resistance to NK cells (Fig. 2). Although cells infected with strains AD169 or Towne are vulnerable to NK cell attack [24–26], the Towne strain became substantially more resistant following repair of the U_L_/*b*′ region (Fig. 2). Thus, AD169 and Towne have clearly lost NK cell evasion functions in this region [27].

We were interested in the complex interaction between HCMV and the host immune response, particularly in characterizing the multiple mechanisms by which the virus systematically evades NK cell recognition. To this end, we set out to establish a system to screen the entire genetic content of HCMV in functional assays by expressing all canonical HCMV protein-coding genes using a bespoke, high-efficiency adenovirus (Ad) vector [28]. This objective required a reliable source of wild-type HCMV genes. As explained above, the genomes of isolated strains degenerate with passage. This issue was less acute with low-passage strains (e.g. Toledo), yet even limited growth in vitro results in altered tropism and the rapid selection of virus that is less cell associated. In order to be able to trust the sequence of a gene within a passaged strain, it was important to compare its sequence with that of the clinical sample from which it was derived. In the absence of any reliable source of HCMV genes amongst the available laboratory or passaged strains, we were obliged to go back to source—a clinical sample.

## Development of strain Merlin

Five neonatal urine samples diagnosed positive for HCMV by PCR were kindly provided by Public Heath Laboratories (PHLS/NPHS), Cardiff. The viruses were amplified in fibroblast cell culture to generate sufficient DNA for shotgun cloning into an M13 vector and Sanger sequencing. Strain Merlin (clinical sample 742) was prioritized on the basis of its efficient recovery from frozen (−70 °C) stocks and genomic integrity in preliminary sequencing analyses. Each passage of Merlin involved the serial infection of an uninfected fibroblast monolayer with cell-free supernatant. The complete genome sequence of Merlin was determined from virus at passage 3, and the gene content was annotated [29, 30]. At the time, this was the first complete HCMV genome sequence to be determined, and this resulted in Merlin being designated as both the NCBI RefSeq standard [31] and the first World Health Organization (WHO) International Standard for HCMV [32].

At least one genetic change was already evident in the Merlin genome sequence by passage 3, and further deterioration would be inevitable with further culture. A solution to this problem was provided by bacterial artificial chromosome (BAC) cloning [33, 34]. BACs are low copy number plasmids compatible with the cloning, maintenance and manipulation of large DNA fragments in *Escherichia coli.* Not only can the HCMV genome be maintained in *E*. *coli* without accruing further mutations, but the technology also provides a robust source of clonal, genetically defined virus and greatly facilitates manipulation of the viral genome. Multiple HCMV strains had previously been BAC cloned, including the high-passage strains AD169 [33, 35] and Towne [36–38], as well as the low-passage strains Toledo, PH, TR [39], FIX [40] and TB40/E [41]. However, none of these constructs was suited to our purpose. Except for one BAC based on AD169 [35], all constructs incorporated the vector cassette as a stably integrated element within the U_S_ region, where it replaced genes US2, US3, US6 and (in some cases) US11. Consequently, viruses derived from these BACs do not regulate MHC-I or MHC-II in the same manner as clinical virus, and this has profound effects on NK and T cell assays. Moreover, since the original clinical material appeared not to be available for any of these BACs, the extent to which any of them accurately represented clinical virus could not be determined. We and others have since shown that these clones contain both obvious and subtle mutations that were probably acquired in vitro prior to BAC cloning and that these changes impact viral tropism [42] and interactions with NK cells [43].

To secure a reliable, definitive source of wild-type HCMV genes, the complete genome of Merlin, from DNA harvested at passage 5, was inserted into a BAC plasmid [44]. To make it possible to derive virus containing the complete genome from the BAC, the vector cassette was designed to be self-excising using Cre/LoxP recombination, as had been done previously for pseudorabies virus [45] and HCMV [35, 46]. As a result, virus derived from the BAC by transfection does not contain the vector cassette and differs from the parental genome at this locus merely by the presence of a 34-bp LoxP site following gene US28.

This BAC provided a reproducible source of clonal virus and enabled seamless manipulation of the viral genome by using DNA recombineering [44]. Sequencing of the prototype Merlin BAC clone identified a nucleotide substitution in UL128 that was known to have been selected during the first passage of Merlin in vitro [18, 29]. Sequencing of multiple clones further showed that all were also mutated in RL13, but that not all mutations were the same. The viral population prior to BAC cloning must therefore have contained a single mutation in UL128 and a variety of mutations in RL13. The original BAC was therefore repaired to match the presumed sequence in the clinical sample, except for three non-protein-coding differences in the *b*/*b’* region. We have since sequenced Merlin directly from the clinical sample (which, crucially, had been retained) and found that, apart from these minor variations in the *b*/*b’* region and the inserted *Lox*P site, the repaired Merlin BAC matches the clinical virus (unpublished data).

**Fig. 3:**
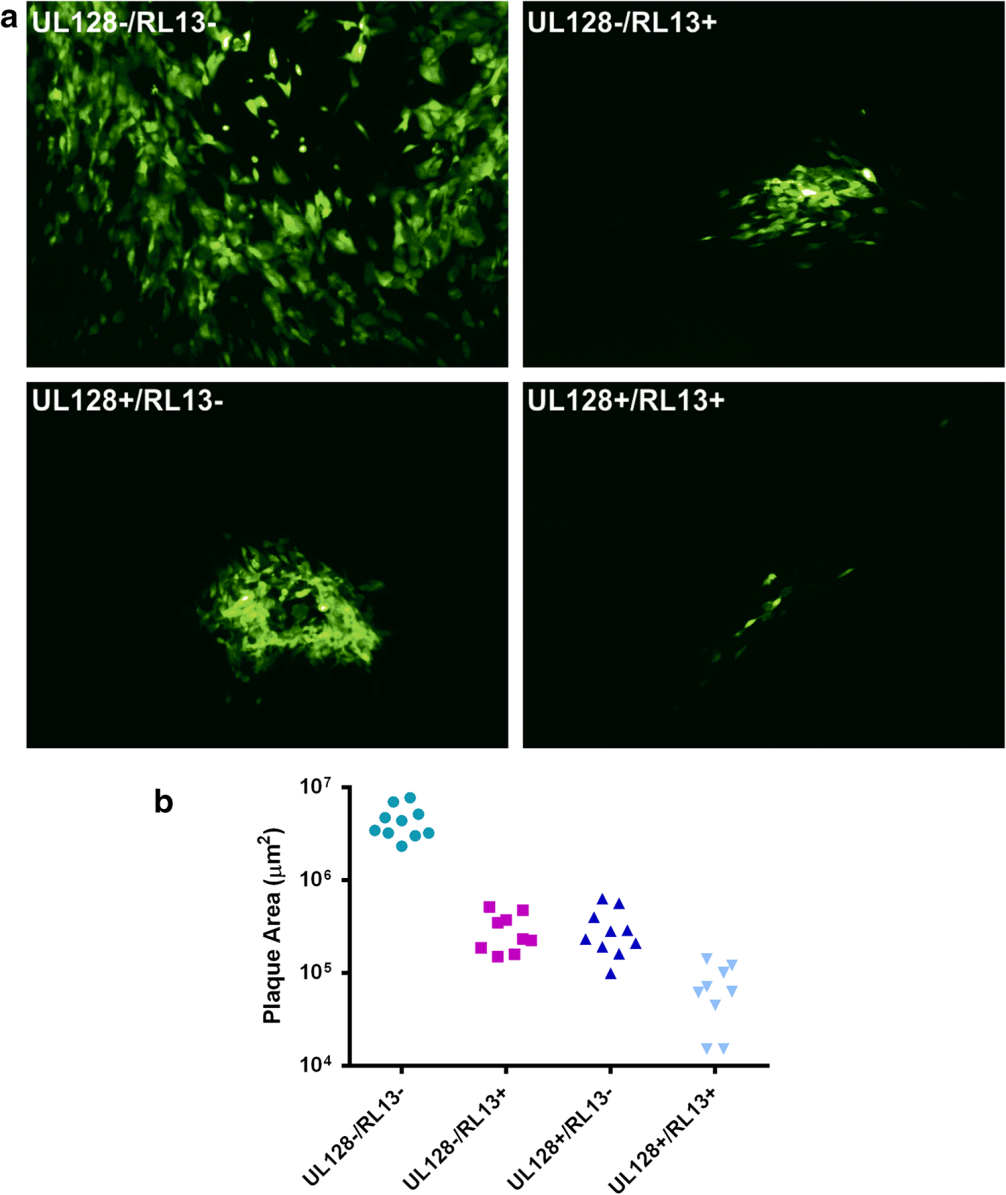
Impact of RL13 and UL128L on HCMV replication. Fibroblasts were transfected with Merlin BAC constructs in which either (or both) RL13 and UL128 were mutated. **a** Plaques in fibroblast monolayers were readily visualized at 3 weeks post-transfection using an eGFP reporter function. **b** Areas of individual plaques measured at 3 weeks post-transfection. Cells were grown under an overlay to prevent cell-free spread of virus. Adapted from figures originally published in *Journal of Clinical Investigation* [44], reusing the author’s own content with permission

Virus generated from Merlin BAC constructs in which either UL128 or RL13 had been repaired exhibited a markedly reduced capacity to replicate in fibroblasts as overtly manifested by a reduced plaque size (Fig. 3). Repair of both genes had an even more profound effect on viral growth, implying the two genes impair viral replication by distinct mechanisms. Independent mutations in RL13 and UL128L were rapidly selected in a manner similar to that observed when passaging clinical isolates [17, 44]. RL13 encodes a virion glycoprotein, but its role in virus replication is currently unclear [44]. Along with gH/gL, the UL128L proteins form a pentameric virion envelope glycoprotein complex that promotes infection of endothelial, epithelial and myeloid cells [19, 47–51], yet impedes efficient replication in fibroblasts [17, 44]. However, in vitro propagation of viral stocks with a wild-type gene complement was achieved by using selective repression to inhibit expression of both RL13 and UL131A [44].

The Merlin BAC contains a complete HCMV genome that is thought accurately to represent the original clinical agent from which it was derived. It is also a reproducible source of clonal virus (via transfection), is capable of reconstituting phenotypically wild-type virus, and is suitable for rapid manipulation of the viral genome by recombineering technology [44]. Consistent with our primary objective, all canonical genes have now been sub-cloned into an adenovirus vector for use in functional screening (unpublished data). This knock-in approach has been complemented by a knock-out system, in which a series of HCMV recombinants has been generated, each deleted in a specific block of genes [52]. The two strategies combine well and have already enabled the identification of three novel immune evasion genes in HCMV [52, 53].

## Systems biology and HCMV

Confidence in the genomic integrity of Merlin has underpinned the application of high-throughput technologies to study the virus and virus-infected cells. Next-generation sequencing has been used to compare passaged derivatives with virus in clinical samples and routinely to assess the genetic integrity of Merlin BAC constructs and their viral progeny. RNA-Seq, combined with conventional RNA mapping studies, has revealed that long non-coding RNAs (lncRNAs) make up >65 % of polyadenylated HCMV RNA produced in fibroblasts and that the levels of splicing and expression of antisense RNAs are far more extensive than suspected previously [54]. Annotation of the HCMV genome currently identifies 170 canonical protein-coding genes plus four lncRNAs. A high-definition analysis of the HCMV transcriptome aided by ribosome profiling predicted 751 translated open reading frames (ORFs); albeit 245 are <20 codons in size and a substantial number commence with non-conventional initiation codons or are internal to, or overlap, the canonical ORFs [55]. The expression of a subset of these putative novel proteins has been detected by mass spectrometry and epitope tagging. Moreover, preliminary studies have detected CD4+ and CD8+ T cell responses to proposed novel, small proteins encoded by an exceptionally high abundance lncRNA (β2.7 or RNA2.7), providing further evidence for the existence of these proteins during natural infection [56, 57]. The full extent to which non-canonical ORFs impact the biology of HCMV has yet to be determined.

**Fig. 4:**
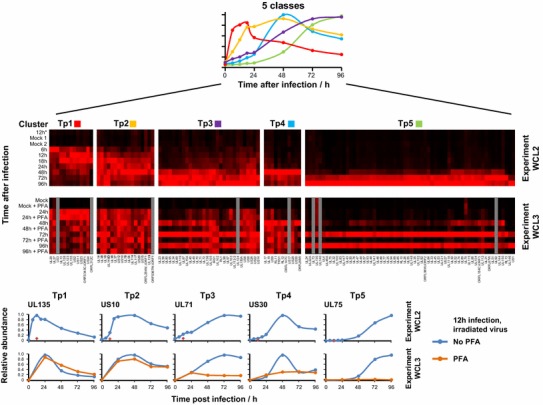
Temporal classification of HCMV gene expression. QTV was used to track the expression of 139 canonical and 14 non-canonical genes through productive infection of fibroblasts by HCMV strain Merlin. Distinct profiles emerged when gene expression was separated into as few as five temporal classes (Tp1–5). Adapted from a figure originally published in *Cell* [58], reusing authors own content

In addition to engaging in transcriptome analysis, we have developed quantitative temporal viromics (QTV) as a proteomics-based approach for following productive infection of fibroblasts with Merlin [58, 59]. The opportunity was also taken to analyse the effects of disabled (irradiated) input virions, as well as those of an inhibitor of viral DNA replication. QTV compared the expression of >8000 proteins in the whole cell and 1184 proteins at the cell surface, in order to provide the most detailed analysis to date of a virus-infected cell. QTV also tracked the expression of 139 canonical and 14 non-canonical HCMV proteins through the course of infection. This resource has already provided insights into the manipulation of signalling pathways and immune defences [58]. Furthermore, it provided an opportunity to examine the temporal cascade of viral gene expression. The division of herpesvirus gene regulation into immediate-early, early and late phases based on the application of metabolic inhibitors is convenient, yet artificial. An unbiased, computer-based analysis of HCMV protein expression by QTV indicates that the cascade of gene expression can be most effectively divided into five temporal classes (Tp1–5), to which most HCMV canonical genes have been assigned (Fig. 4). Cutting-edge technologies are thus bringing extreme definition to our understanding of how this most complex of human viruses regulates both host and viral gene expression.

## HCMV strain usage and terminology

As described above, clinical HCMV strains must acquire specific mutations in order to replicate efficiently in vitro. Weller observed that the “serial propagation of the cytomegaloviruses characteristically results in the production of cell-free virus in higher titre” [60]. Indeed, human vaccine trials using Towne and AD169 indicated that these strains are attenuated extensively and are eliminated rapidly in vivo [61–63]. Waldman and colleagues characterized the loss of both endothelial cell tropism and cell association during culture in vitro [64, 65]. These phenotypic changes were shown to be due to genetic alterations occurring in vitro [66] and were ultimately found to be due, at least in part, to the acquisition of mutations in RL13 and UL128L [17, 19, 44, 47–50, 67, 68]. Thus, as the virus is cultured in vitro, the virion envelope loses the RL13, UL128, UL130 and UL131A proteins, and, eventually, other components. These changes impact dramatically not only the growth properties and tropism of the virus, but also its sensitivity to neutralizing antibodies, since the UL128L proteins are a major target of neutralizing antibodies in vivo [69–75]. Indeed, HCMV vaccine studies are now strongly focused on the UL128L proteins [69, 76–82]. A clear lesson from these developments is the need to ensure that the HCMV strain under study is clinically relevant. On the 60th anniversary of the first isolation of HCMV, it is appropriate to reassess the nature of the viral strains that are currently being used to study HCMV tropism and pathogenesis and to develop urgently needed antiviral and immunotherapeutic treatments.

What are the most appropriate terms for the viruses with which we are working? The term *laboratory strain* is normally reserved for AD169 and Towne. Although these strains have had a major impact on HCMV research, their genomic integrity has suffered so dramatically through extensive passage in vitro that they should not be considered as adequate representatives of the causative agent of clinical disease. Great caution needs to be taken in interpreting the findings made using them, particularly in studies of tropism and pathogenesis. As a result of these considerations, we elected not to use laboratory strains to screen for NK cell modulatory functions but to develop Merlin as a source of wild-type HCMV genes. Although a subset of NK modulators (UL16, UL18 and UL40) were identified by using AD169, at least three additional examples (UL135, UL141 and UL142) are known to have been deleted from both laboratory strains (reviewed in [21, 83]). Moreover, mutations that potentially impact NK cell recognition have been detected in UL40 in strains Towne, TB40/E and U8, and in UL141 in AD169, Towne, TB40/E and VR1814 [15, 17, 29, 43] (Table 1). It is not clear whether the functional defect in the HLA-E binding peptide encoded by UL40 in TB40/E is a natural variant or whether it was acquired in vitro [43]. What is clear is that defects in immune evasion functions tend to accumulate with increased passage number.

**Table 1:** HCMV NK evasion functions

Gene	Target	Conserved
UL18	HLA-1 homologue, binds LIR1	Yes (variable)
UL40	Upregulates HLA-E and gpUL18	Mutation in TB40E, Towne, U8
UL16	MICB, ULBP1, ULBP2	Yes
UL83	Binds NKp30	Yes
miR112	miRNA against MICB	Yes
UL135	Inhibits synapse formation	Deletions in AD169, Towne
UL141	PVR, Nectin 2 TRAIL-R	Deletions in AD169, Towne, TB40E VR1814, some low-passage strains
UL142	MICA	Deletion in AD169, Towne, some low-passage strains
US18	MICA	Yes
US20	MICA	Yes

## Perspective

HCMV isolates other than *laboratory strains* tend to be designated *clinical* or *low*-*passage strains* by default. The term *clinical* is uninformative, as all HCMV strains were derived originally from clinical material. *Lowpassage* is potentially a more useful term, yet it is elastic and frequently used to refer to viruses that have been passaged quite extensively in diverse cell types. Furthermore, even if a virus was *lowpassage* originally, it will become *highpassage* as it is grown and will inevitably adapt further. Ambiguities arising from findings made by using undefined reagents are clearly unhelpful, and there is merit in using a more precise terminology to describe and define particular strains. We suggest that it may be prudent to reserve the term *clinical strain* in publications for a virus that has not been passaged in vitro (i.e. the virus in the clinical sample), and to support the use of *low*-*passage strain* by details of provenance, passage number, nature of passage (via infected cells or cell-free virus) and cell type used for passage. However, what matters most is the genetic state of the virus, and this is much more evident from sequence analysis than it is from passage history. Even then, the sequence of any passaged strain needs to be compared with that in the clinical sample, in order to identify mutations that have been selected during in vitro adaptation.

Research into a virus should involve the use of a strain that represents the clinical agent as closely as possible. In this context, HCMV research is in a cleft stick. On the one hand, although low-passage HCMV strains may transiently be wild type, they are highly cell associated and therefore cannot readily be used in functional assays [17]. On the other hand, passaged virus is less cell associated and is therefore more tractable, but is not likely to be wild type. A degree of compromise is necessary in order to make experimentation possible. The use of passaged strains (that are known to be compromised) will continue to be necessary for certain applications. However, the limitations of such studies should be recognized. For example, the high titre and broad tropism of strain TB40/E (and viruses derived from its BAC clone, TB40-BAC4) are invaluable when conducting studies requiring efficient infection of myeloid, endothelial or epithelial cells. However, the exceptional properties of this strain [41, 42] should caution against viewing findings as necessarily being true of HCMV generally. Crucially, a capacity to reference the clinical sample would remove uncertainty about the integrity of passaged HCMV strains in relation to the original virus from which they were derived. More generally, the efficient propagation of viral stocks appears to require only that expression of RL13 and UL128L be ablated or suppressed (e.g. by subtle mutation [42] or overt repression [44]). In these circumstances, RL13 and UL128L expression is suppressed specifically to alter the biological properties of the virus, and thus is no longer wild type. Nevertheless, this compromise does allow researcher to migrate towards adopting a genetically reliable HCMV strain.

When the genome sequences of HCMV strains are compared, a remarkably high level of variation is evident. This is uneven across the genome [29] and is at its most extreme in a group of hypervariable genes (e.g. RL12, RL13, UL74, UL146 and UL139) in which different genotypes can exhibit as little as 38 % amino acid sequence identity [84–90]. Some unpassaged viruses have also been shown to carry mutations likely to result in the loss of certain gene functions (e.g. RL5A, RL6, UL1, UL9 and UL111A) [91, 92]. Identical mutations have been observed in geographically distinct strains, implying that strains mutated in certain canonical genes are circulating in the population. Furthermore, deep sequencing has suggested that the virus may evolve in vivo on much shorter timescales than previously appreciated [93, 94]. If this is true, at least a proportion of this heterogeneity can be expected to impact viral gene function and pathogenesis.

These observations probably reflect the complex relationship that the virus has with the host immune system, heterogeneity in the virus being required to enable it to cope with heterogeneity in the host [95, 96]. They also imply that use of one or a small number of viral strains in HCMV research, even if the reagents are well designed and carefully monitored, is unlikely to provide an adequate view of the biology of HCMV. High-throughput technologies (genomics, transcriptomics and proteomics) that have rapidly and comprehensively informed on infection with strain Merlin could readily be applied to studies of HCMV strain variation. Although Merlin has several features that commend it as a strain for general use in research, there is also a need to recognize the natural diversity of HCMV. To enable such studies, there would be clear merit in constructing a substantial set of BAC clones that each contains a complete viral genome matched to the sequence in the clinical sample.
